# Baseline assessment of microplastic contamination in agricultural soils from the coastal stretches of Karnataka and Goa, Southwestern India

**DOI:** 10.1007/s10661-025-14513-5

**Published:** 2025-08-28

**Authors:** Lohani Mahreen, Ashwathi C, Anish Kumar Warrier

**Affiliations:** 1 Department of Sciences, Manipal Institute of Technology, Manipal Academy of Higher Education, Manipal, Udupi, Karnataka 576104 India; 2 Centre for Climate Studies, Department of Civil Engineering, Manipal Institute of Technology, Manipal Academy of Higher Education, Manipal, Udupi, Karnataka 576104 India

**Keywords:** Soil pollution, Agroecosystems, Fibres and fragments, Paddy fields, Soil Management

## Abstract

Microplastic (MP) contamination in terrestrial ecosystems—particularly in agricultural soils—remains an underexplored environmental concern in India, an agriculture-driven economy. This study assessed MP contamination in paddy fields across 15 sites in Udupi (Karnataka) and Goa, with soil samples collected from the surface and at three subsurface depths (10, 20, and 30 cm). MPs were extracted using density separation, identified via stereomicroscopy and ATR-FTIR, and further characterized using SEM–EDS to examine surface morphology and elemental composition. Microplastics were detected in all samples, with higher concentrations observed in Goa across most depths. In Goa, surface soils contained an average of 100.93 ± 64.19 pieces/kg, while subsurface layers at 10, 20, and 30 cm recorded 90.70 ± 40.37, 119.76 ± 169.44, and 48.34 ± 29.75 pieces/kg, respectively. In comparison, Udupi samples exhibited slightly lower concentrations, with 95.68 ± 30.69 pieces/kg at the surface, and 55.00 ± 29.33, 46.53 ± 9.04, and 16.10 ± 7.63 pieces/kg at corresponding depths. Shape analysis revealed regional differences: in Udupi, fibres dominated (86.8%), followed by films (10.3%) and fragments (3.0%), whereas in Goa, fibres were less dominant (50.8%), with a higher proportion of films (36.7%) and fragments (12.0%). Black-coloured MPs and polypropylene polymers were most prevalent overall. SEM analysis indicated signs of environmental weathering, and EDS confirmed the presence of toxic heavy metals (Fe, Cd, and As) on MP surfaces. The highest Polymer Hazard Index (PHI) value of 1859.9 was recorded in Goa, indicating Hazard Level V due to the presence of high-risk polymers such as polyamide and polystyrene. The Coefficient of Microplastic Impact (CMPI) analysis showed that fibre-shaped MPs in Udupi had values > 0.8, indicating strong ecological risk, while Goa exhibited a more balanced morphology impact (CMPI: 0.11–0.50). These findings underscore the widespread presence of MPs in agricultural soils and highlight the urgent need for routine monitoring and the development of mitigation strategies to protect soil health and food security.

**Supplementary Information:**

The online version contains supplementary material available at 10.1007/s10661-025-14513-5.

## Introduction

Microplastic (MP) particles, defined as plastics  ≤ 5 mm in size, have emerged as pervasive environmental contaminants, posing significant threats to both terrestrial and aquatic ecosystems (Thompson et al., 2004). While MPs have been extensively studied in marine and freshwater environments, their presence and impact in terrestrial ecosystems—particularly in agricultural soils—remain relatively underexplored (Amaneesh et al., 2025). Agriculture plays a vital role in India’s economy, with the country ranking among the world's leading producers of staple crops such as wheat, pulses, and rice (Food and Agricultural Organization, 2024). The intensive nature of agricultural practices, especially in rural and coastal areas, renders Indian farmlands increasingly susceptible to environmental contaminants, including MPs.

Microplastics can enter agricultural soils through multiple pathways, including the application of sewage sludge, the use of compost or organic fertilizers contaminated with plastic residues, plastic mulch films, irrigation with wastewater, atmospheric deposition, and runoff from nearby urban or industrial areas (Nizzetto et al., 2016; Qi et al., 2018). Once introduced, MPs can persist in the soil for extended periods due to their resistance to degradation (de Souza Machado et al., 2018). Their presence may alter soil structure, disrupt microbial communities, reduce water retention capacity, and negatively affect plant growth (Gao et al., 2025; Hasan & Tarannum, 2025). Furthermore, MPs in soil raise concerns about their potential uptake by crops, posing risks to food safety and human health (Yu et al., 2022).

To date, most research on MP pollution in agricultural soils has been conducted in China (Wang et al., 2022; Zhou et al., 2024). These studies cover a wide range of agricultural systems, including rice paddies, vegetable farms, and intensively cultivated farmlands (Liu et al., 2018; Wang et al., 2022; Yang et al., 2023) (Table 1). In contrast, studies from India remain limited (Borah et al., 2024; Ranjani et al., 2021; Singh et al., 2024a, b), and have predominantly focused on MP contamination in river water, sediments, and lacustrine ecosystems (Amrutha & Warrier, 2020; Amrutha et al., 2023; Sarkar et al., 2020; Warrier et al., 2022). This highlights a significant gap in foundational research on MPs in agricultural soils along India's coastal stretches. The absence of comprehensive baseline data hampers the assessment of contamination levels, the monitoring of spatio-temporal trends, and the development of effective mitigation strategies (Singh et al., 2024a, b). Establishing such baseline information is essential for addressing MP pollution in terrestrial agricultural systems and safeguarding long-term soil health and food security.

**Table 1:** Summary of selected studies on MP contamination in paddy field environments

References	Location	MP concentration	Shape	Polymer types
Wang et al., 2022	Xiangtan City, Southern China	3805 ± 511 n/kg	Films	PVC
Yang et al., 2023	Southwest China	76.2 ± 18.4, 118.6 ± 44.8, and 159.6 ± 23.5 items/kg	Films	Polyesters, polyethylene
Corradini et al., 2019	Chile	1.1 to 3.5 particles/g dry soil	Fibre	Polyester
Singh et al., 2024a	Bhauri and Kokta, Bhopal	307.5 ± 9.19 and 69.5 ± 4.95 particles per kg	Fibre, fragments	Polypropylene, polyethylene
Karthika et al., 2024	Ooty and Coimbatore, Tamil Nadu	paddy field (1,500 items/kg)	Fragments, fibre	Polyethylene
Present study	Goa and Udupi, India	Goa- 100.93 ± 64.19 pieces/kg Udupi-95.68 ± 30.69 pieces/kg	Fibre, Films, Fragments	Polypropylene, Polyethylene

This study was conducted to generate baseline data on MP pollution in the agricultural soils of Goa and Udupi, Karnataka, in light of the significant research gap in this area. It aims to assess the prevalence of MPs in soil, with a focus on their morphological attributes—such as shape, colour, and size—as well as their polymer composition. Surface features of selected MP particles were examined using scanning electron microscopy (SEM) to identify degradation patterns and infer potential sources. This research aligns with several United Nations Sustainable Development Goals (SDGs), notably: SDG 2 (Zero Hunger), by addressing threats to soil health and food security; SDG 3 (Good Health and Well-being), by identifying potential human exposure pathways; SDG 12 (Responsible Consumption and Production), through the investigation of plastic residues in agricultural systems; and SDG 15 (Life on Land), by supporting the conservation and sustainable use of terrestrial ecosystems.

### Study area

**Fig. 1:**
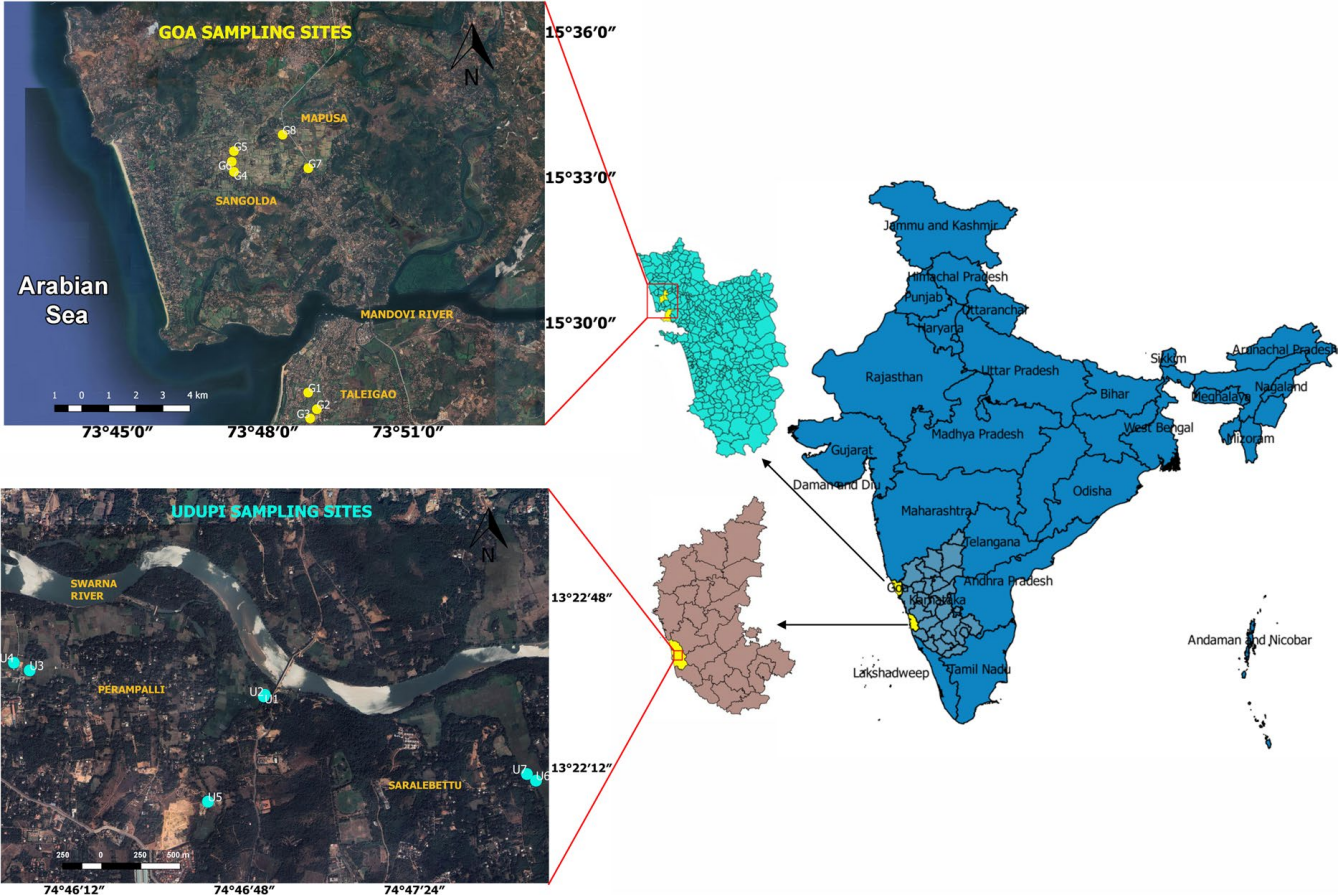
Map showing the sampling locations selected for the study. A total of 15 agricultural soil sampling sites were investigated, comprising eight sites in Goa and seven sites in Udupi, Karnataka. All sampling locations are situated within active paddy rice fields, representing typical agricultural land use in the region

This study on MP pollution in agricultural soils was conducted in Udupi (Karnataka) and Goa, located along India's southwestern coast (Fig. 1). Goa, the smallest Indian state, is bordered by Maharashtra to the north, Karnataka to the east and south, and the Arabian Sea to the west. The Western Ghats—a prominent mountain range and recognized biodiversity hotspot—run along Goa’s eastern boundary. Udupi, a coastal district situated just south of Goa, lies within Karnataka. In both regions, rice is the staple crop, and paddy cultivation forms a central component of local agriculture (Sonak, 2014; Vaz et al., 2017).

Goa receives an average annual rainfall ranging from 2500 to 4500 mm, with approximately 90% occurring between June and September (Singh et al., 2017). Udupi, by comparison, receives even higher annual rainfall—ranging from 3800 mm to over 4000 mm (Deepika et al., 2020). Both regions experience a humid tropical climate; however, Udupi is characterized by more intense agricultural land use, especially in the form of plantations and rice paddies (India Meteorological Department, 2023; Karnataka State Natural Disaster Monitoring Centre, 2023). Goa, on the other hand, is subject to greater urbanization and tourism-related pressures, with dense coastal development.

The agricultural soils in Goa are predominantly lateritic in the highlands and alluvial in the lowlands, while Udupi soils are mainly alluvial and clayey. A total of 15 agricultural fields were selected for sampling—eight sites in Goa and seven in Udupi (Fig. 1). In Goa, sampling was conducted in Taleigao, Sangolda, and Mapusa, where paddy is grown during the monsoon using rainwater, followed by the cultivation of vegetables such as green spinach, red spinach, sweet potatoes, and black-eyed beans. The Goan sites were located near busy roads and rural settlements, suggesting increased exposure to anthropogenic sources of contamination.

In contrast, the Udupi sampling sites were located near the banks of the Swarna River, specifically in the villages of Perampalli and Saralebettu. These fields are more remote than the Goan sites and are exclusively dedicated to paddy cultivation, relying primarily on rain-fed irrigation. In one field, a small canal was observed supplying water from the Swarna River to the paddy fields.

## Materials and methods

### Sampling

Soil sampling was conducted in December 2024 from paddy fields in Goa and Udupi. At each site, soil was collected from the surface layer and three subsurface depths: 0–10 cm, 10–20 cm, and 20–30 cm. Depth measurements were verified using a stainless-steel ruler. For surface sampling, five subsamples were collected per field—one from each corner and one from the centre—and thoroughly homogenized to form a single composite surface soil sample (Yu et al., 2021). For subsurface layers, three trenches were excavated diagonally across each field to access the designated depths. At each depth, soil was homogenized to produce one composite sample. All samples were collected in clean aluminum containers, properly labeled, and transported to the laboratory for further analysis. Field blanks were also prepared by exposing pre-cleaned filter paper to ambient air at the sampling site to estimate potential airborne MP contamination during sample collection.

### Sample processing

A total of 400 g of soil was collected from each agricultural field and air-dried at 60 °C until all moisture had evaporated. The dry weight of each sample was then recorded. To disperse soil particles, 300 mL of sodium hexametaphosphate solution was added to each sample (Bottone et al., 2022), and the mixtures were agitated on a rotary shaker at 250 rpm for one hour. Following dispersion, 300 mL of zinc chloride (ZnCl₂; 933.3 g/l)) solution was added for density separation, and the mixtures were left undisturbed for 24 h to allow settling (Mahdavi et al., 2022). The settled material was sequentially sieved through 5 mm and 100 µm meshes. Particles > 5 mm were collected in zip-lock bags for further analysis, while the fraction between 5 mm and 100 µm was transferred into 500 mL glass beakers and dried at 60 °C. To remove organic matter, 30 mL of hydrogen peroxide (H₂O₂) was added to each dried sample and left to react for 48 h (Prata et al., 2019). Residual hydrogen peroxide was evaporated by gentle heating on a hot plate. A second density separation was then performed by adding 150 mL of ZnCl₂ solution, followed by another 24-h settling period. Finally, the mixtures were sieved through 1 mm, 300 µm, and 100 µm meshes (Valsan et al., 2024). The retained fractions were placed in glass Petri dishes, thoroughly dried, and stored for MP identification.

### Microplastic identification

Following extraction, samples were examined under a Nikon SMZ745T stereo zoom microscope. Microplastic particles were identified based on distinctive physical features, including shape, size, and colour, and were digitally photographed. Larger MP particles were separated and stored in labelled zip-lock bags for polymer identification using Attenuated Total Reflectance Fourier Transform Infrared Spectroscopy (ATR-FTIR) (Thakur et al., 2023). Spectra were recorded over the range of 400 to 4000 cm⁻^1^. A total of 92 MP particles within the size ranges of 1–5 mm and 0.3–1 mm were analyzed. In addition, Scanning Electron Microscopy (SEM) was used to examine the surface morphology of four representative MP particles, providing insights into weathering and degradation features (Li et al., 2020). Three MP films in the 1–5 mm size range were also analysed.

### Quality assurance and quality control

To minimize contamination during sampling, both surface and subsurface soils were collected using a wooden spatula and stored in clean aluminum containers sealed with aluminum foil. Plastic tools were strictly avoided. During both field and laboratory work, cotton lab coats and nitrile gloves were worn. All laboratory equipment was thoroughly cleaned with distilled water, and glassware was kept covered with aluminum foil. Work surfaces were regularly cleaned to reduce airborne or contact contamination. A total of eight procedural blanks were included to detect any contamination introduced by laboratory equipment or reagents. To assess extraction efficiency, recovery tests were conducted using two soil samples each from Goa and Udupi, spiked with a known number of MPs (20 fibres, 15 foams, 15 fragments, and 15 films; total = 65 particles per sample). The final recovery rate was 98.08%, confirming the reliability and effectiveness of the extraction procedure.

### Statistical analysis

To determine the differences in MP abundance across surface and subsurface layers, a one-way analysis of variance (ANOVA) was performed using Microsoft Excel. To evaluate differences in MP abundance between locations and across soil depths, a two-way analysis of variance (ANOVA) was conducted, with location and depth as fixed factors. Following ANOVA, Tukey’s Honest Significant Difference (HSD) post hoc test was applied to assess pairwise differences between groups. All statistical analyses were performed using R (Version 4.4.2). Geospatial mapping of the sampling locations and study area was carried out using QGIS (Version 2.18.5). Graphs and visual representations of the results were prepared using Grapher (Golden Software, Version 16.0).

### Risk assessment

This study also aims to evaluate the pollution risks posed by MPs in agricultural soils, following the approach outlined by Ranjani et al. (2021). Two indices were applied to assess ecological risk: the Polymer Hazard Index (PHI) and the Coefficient of Microplastic Impact (CMPI).

#### Polymer Hazard Index (PHI)

The PHI assesses the potential ecological harm of MPs by considering both their concentration and polymer composition. Since each polymer type has a distinct toxicological profile, a hazard score was assigned to each polymer based on the classification proposed by Lithner et al. (2011). The PHI was calculated as:

$$PHI= \sum Pn \times Sn$$

where P_n_ is the proportion of each polymer and S_n_ is the corresponding hazard score. This index facilitates the evaluation of the relative toxicity risk associated with the polymeric composition of MPs at each site. Based on the calculated PHI values, MPs are classified into five hazard levels (Ranjani et al., 2021): Level I (low risk: 0–1), Level II (moderate risk: 1–10), Level III (high risk: 10–100), Level IV (very high risk: 100–1000), and Level V (extremely high risk: > 1000). 

#### Coefficient of Microplastic Impact (CMPI)

The CMPI quantifies ecological impact based on the morphological types of MPs, as different shapes (e.g., fibres, fragments, films, foams) may interact differently with the environment and biota. The CMPI was calculated as:

$$CMPI=Specific MP shape\div Total MPs$$

The resulting values were classified into impact categories (Rangel-Buitrago et al., 2021): Low impact: 0.0001–0.10, Average impact: 0.11–0.50, High impact: 0.51–0.80, Extreme impact: 0.81–1.00. This metric provides insights into the dominant MP morphotypes at each site and their potential ecological implications.

## Results and discussion

### Microplastic abundance

**Fig. 2:**
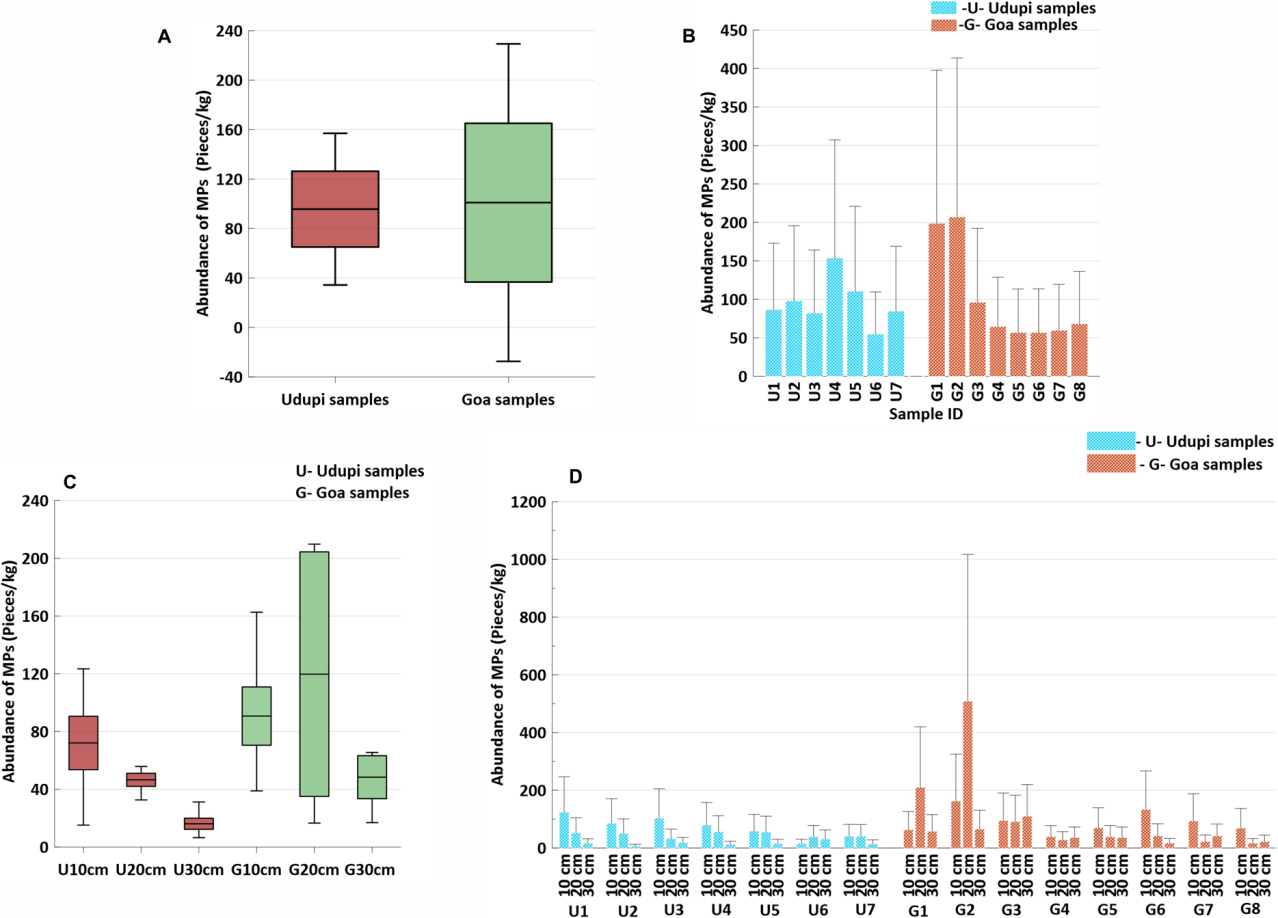
(**A**) Whisker plot showing abundance of MPs in surface soils between Udupi and Goa. (**B**) Abundance graph showing location wise MP concentration in surface soils recorded from Udupi and Goa. (**C**) Whisker plot showing abundance of MPs in soils with respect to depths between Udupi and Goa. (**D**) Location wise MP abundance at three subsurface depths (10 cm, 20 cm, and 30 cm)

Microplastics were detected in all surface and subsurface soil samples from both Goa and Udupi, indicating widespread contamination across agricultural fields in both regions. In Goa, surface samples contained an average of 100.93 ± 64.19 pieces/kg, while subsurface layers at 10 cm, 20 cm, and 30 cm depths recorded 90.70 ± 40.37, 119.76 ± 169.44, and 48.34 ± 29.75 pieces/kg, respectively (Fig. 2). A comparison with other Indian studies underscores the elevated MP abundance in Goa. For example, Singh et al., (2024a, b) reported MP concentrations of 307.5 ± 9.19 particles/kg and 69.5 ± 4.95 particles/kg in 10 soil samples from the Bhauri and Kokta agricultural areas of Bhopal, respectively. While the Bhauri concentration is significantly higher than Goa’s surface average, the Kokta value is comparatively lower. Goa’s MP levels fall between these two extremes, suggesting a moderate to high level of contamination relative to other Indian agricultural sites.

In comparison, Udupi soils showed slightly lower concentrations, with surface samples averaging 95.68 ± 30.69 pieces/kg, and subsurface layers at 10 cm, 20 cm, and 30 cm containing 55.00 ± 29.33, 46.53 ± 9.04, and 16.10 ± 7.63 pieces/kg, respectively. The average MP abundance in Udupi’s agricultural soils was more than double that reported by Borah et al. (2024) for agricultural soils in the Ernakulam district of Kerala (45.6 ± 26.4 particles/kg). The higher abundance in Udupi may reflect greater anthropogenic pressures or differences in land management practices compared to Ernakulam (Borah et al., 2024). The consistent detection of MPs in both surface and subsurface layers points to ongoing contamination from multiple sources, including atmospheric deposition, wastewater irrigation, plastic mulch use, and organic amendments (Corradini et al., 2019; Dris et al., 2016; Zhou et al., 2023). Notably, Udupi consistently displayed lower MP concentrations than Goa (Fig. 2), likely due to Goa's sampling sites being closer to highways, rural–urban interfaces, and human settlements, where anthropogenic activities and atmospheric fallout are more intense.

The maximum surface concentration was recorded at site G2 (Goa). An unexpected increase in MP concentration at the 10–20 cm depth was observed at sites G1 and G2, possibly linked to localized agricultural practices such as deep tillage or irrigation-induced infiltration. A notable diversity of MPs was recorded at 20 cm depth, which may be attributed to localized enrichment at certain sites. In contrast, subsurface samples from Udupi showed a gradual decrease in MP abundance with increasing depth, indicating restricted vertical transport and minimal human disturbance beyond the surface layer (Kumar & Sheela, 2020). This pattern aligns with previous research showing that MP mobility in soils is influenced by factors such as soil texture, rainfall intensity, biological activity, and agricultural intensity.

A one-way analysis of variance (ANOVA) was performed to evaluate the distribution patterns of MPs in surface and subsurface layers across both regions. Surface samples from Goa and Udupi exhibited normal distribution (p ≥ 0.05), indicating a relatively uniform presence of MPs in the upper soil layer. In contrast, subsurface samples displayed geographical differences. In Goa, MP distribution remained consistent with depth, likely due to intensive agricultural practices and proximity to urban and roadside areas, which promote uniform vertical movement. Conversely, Udupi subsurface samples showed a significant deviation from normality (p < 0.05), suggesting spatially uneven MP deposition, likely due to reduced human input and limited vertical mixing in these comparatively undisturbed areas. The presence of MPs in agricultural soils raises concerns about their long-term impacts on soil health, microbial diversity, and crop productivity (Sandil, 2024). These findings highlight the importance of continuous monitoring and sustainable land management strategies to mitigate plastic waste accumulation in terrestrial ecosystems.

A two-way ANOVA was conducted to examine the effects of location (Goa vs. Udupi) and depth (surface, 10 cm, 20 cm, 30 cm) on MP abundance. The analysis revealed marginally significant differences between locations (p = 0.091) and depths (p = 0.073), suggesting potential vertical migration of MPs and regional variation in contamination levels. Although the interaction between depth and location was not statistically significant (p = 0.63), a decreasing trend in MP abundance with increasing depth—particularly in Udupi—was evident. These patterns may reflect differences in anthropogenic pressures and land management practices between the two regions. Post hoc comparisons using Tukey’s Honest Significant Difference (HSD) test were performed to explore pairwise differences; however, none reached statistical significance at the 0.05 level.Wang et al. (2022) investigated MP contamination in rice fields in Xiangtan City, southern China, and reported concentrations of 3805 ± 511 n/kg (Table 1), with films as the predominant morphology and polyvinyl chloride (PVC) as the dominant polymer—differing from the present study’s findings in paddy fields. Similarly, Yang et al. (2023) examined paddy soils in Southwest China exposed to prolonged plastic mulching and found that MP abundance increased over time, with polyester and polyethylene as the predominant polymers. In contrast, studies in India (Karthika et al., 2024; Singh et al., 2024a) reported fragments and fibres as the dominant morphologies, along with higher overall MP abundances than those observed in the current investigation.

### Shape, size, and colour of microplastics

**Fig. 3:**
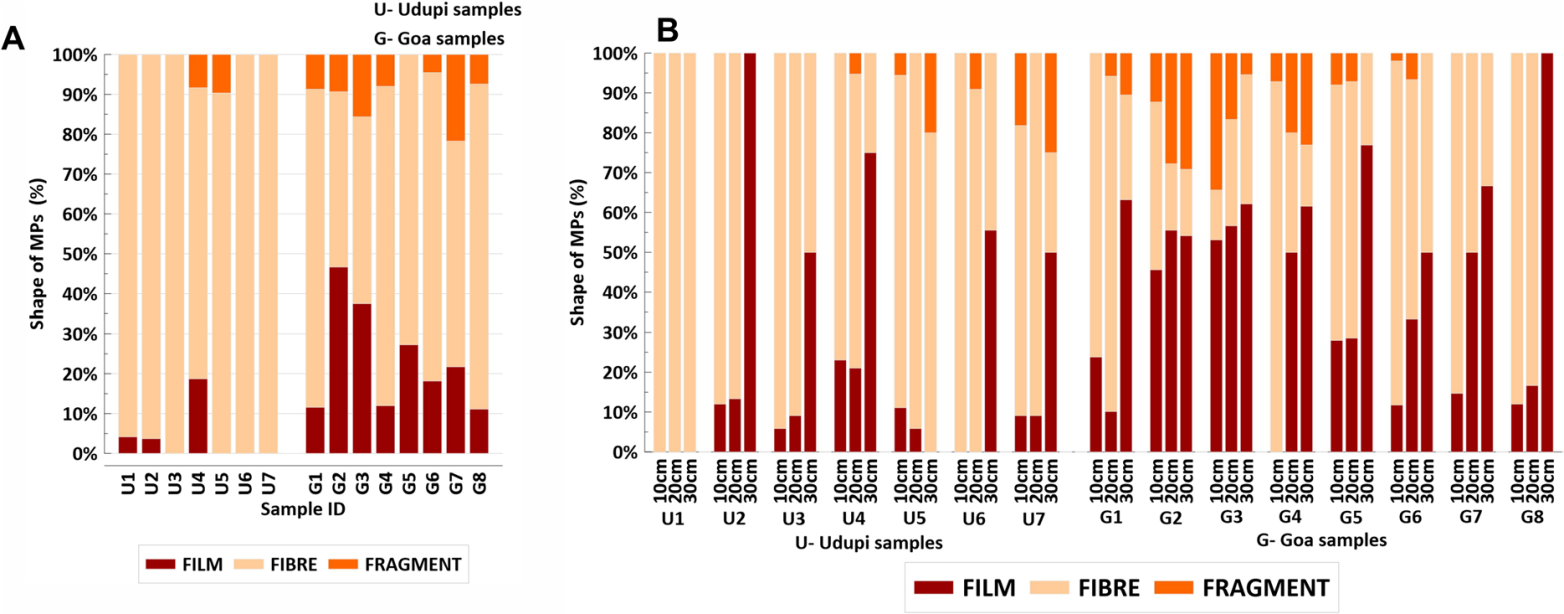
Distribution of MP shapes in agricultural soil samples from Goa and Udupi. (**A**) Percentage composition of different MP shapes in surface samples. (**B**) Percentage composition of different MP shapes in subsurface samples (10–30 cm depth)

Microplastics detected in all examined soil samples were classified into three main morphological types: fragments, fibres, and films (Figs. 3 and 5). Fragments are irregularly shaped particles originating from the breakdown of larger plastic materials; fibres are slender, thread-like particles; and films are thin, pliable sheets of plastic. Among these forms, fibres were the most abundant in both Udupi and Goa. In Udupi, fibres accounted for 86.8% of total MPs, followed by films at 10.3% and fragments at 3% (Fig. 3). In Goa, fibres made up 50.8% of MPs, films 36.7%, and fragments 12.0% (Fig. 3). The predominance of fibrous MPs in this study contrasts with previous research, which reported a higher prevalence of fragmented MPs in agricultural soils (Borah et al., 2024; Shanmugam et al., 2024).

**Fig. 4:**
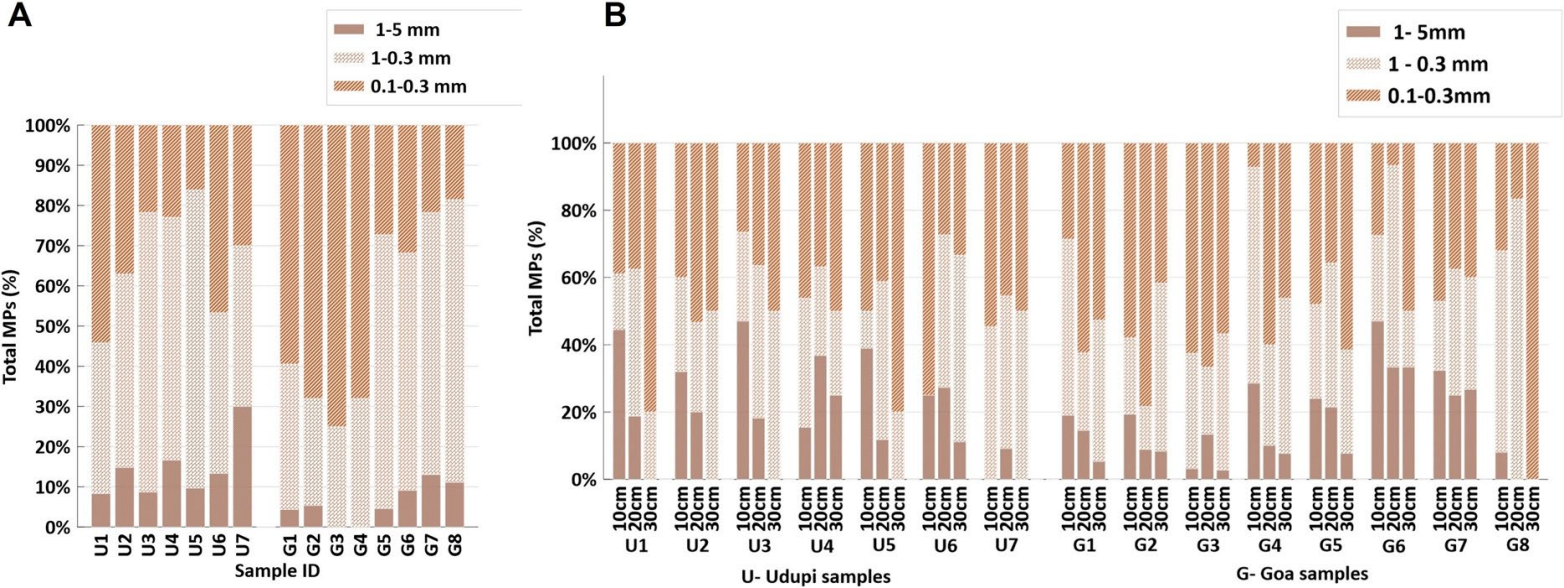
Distribution of MP size classes in agricultural soil samples from Goa and Udupi. (**A**) Percentage composition of different MP size classes in surface soil samples. (**B**) Percentage composition of different MP size classes in subsurface soil samples (10–30 cm depth)

The elevated concentration of fibrous MPs in both regions likely reflects inputs from multiple sources. Wastewater irrigation and sewage sludge applications can introduce fibres shed from synthetic textiles during washing processes (Corradini et al., 2019), while atmospheric deposition can contribute airborne fibres released from clothing, upholstery, and industrial emissions (Hechmi et al., 2024). Fibres can persist in soil for extended periods due to their ability to intertwine with soil particles, potentially altering soil structure and affecting agricultural productivity (Yu et al., 2022). Size distribution analysis revealed that most detected MPs were in the 0.1–0.3 mm range, indicating a predominance of small-sized particles (Fig. 4A, B). The abundance of these smaller MPs may reflect advanced degradation of larger plastic debris under environmental conditions (Corcoran, 2022) as well as their increased persistence in the environment (Thacharodi et al., 2024). Such fine particles are often associated with sources like sewage sludge—where they can escape filtration—and atmospheric deposition, whereby lightweight particles are transported over long distances before settling onto soil surfaces.

**Fig. 5:**
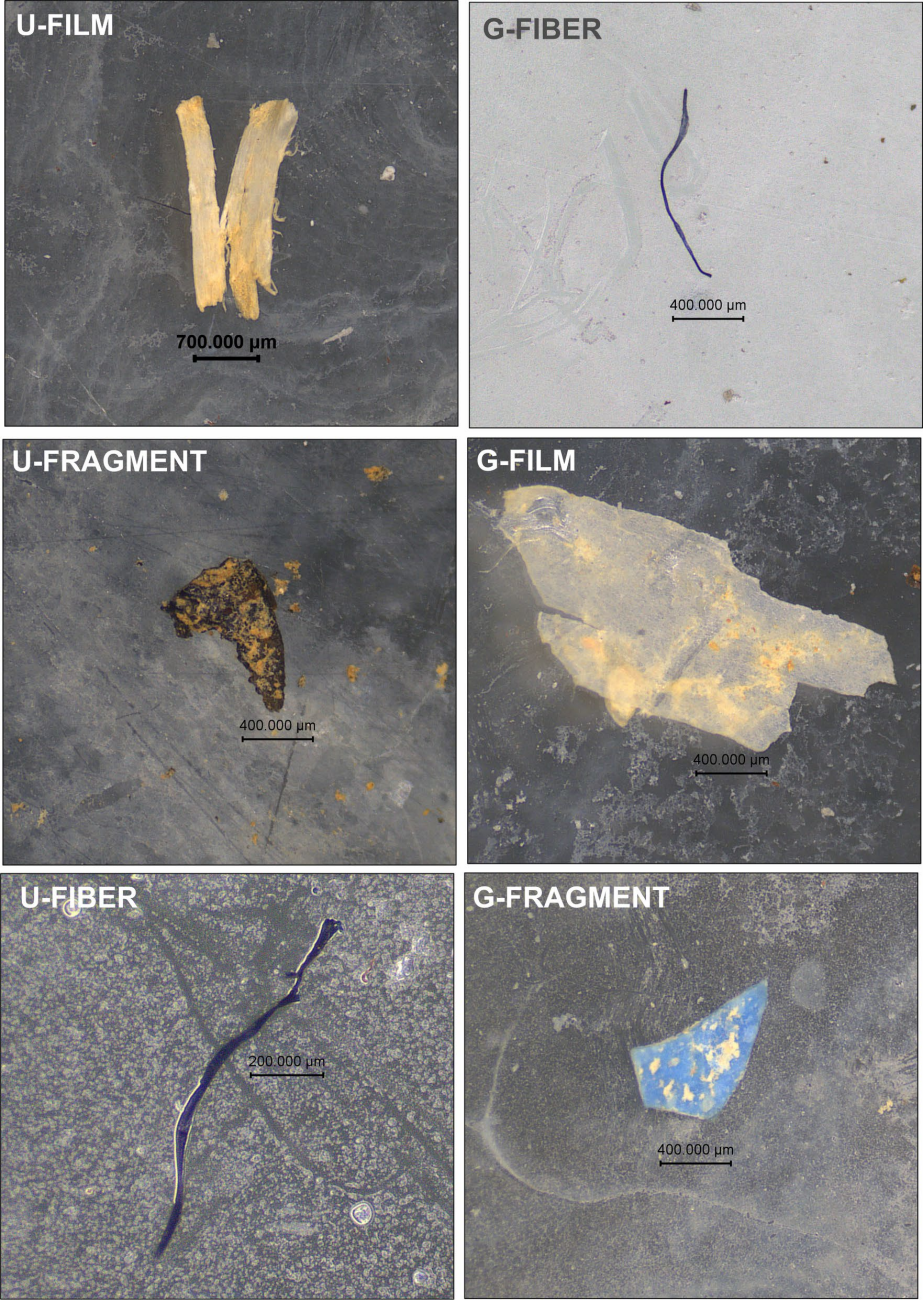
Representative images of MP particles identified in agricultural soils from Udupi (U) and Goa (G)

Microplastics exhibited a varied colour spectrum, with black, blue, transparent, and white being the most frequently recorded across all sampling locations (Fig. 5). The predominance of black particles suggest the widespread presence of dark-hued MPs in the examined agricultural soils. Colour can often indicate potential sources—black and blue particles are commonly linked to synthetic textile fibres, whereas transparent and white MPs are typically associated with plastic packaging materials and agricultural films used in farming and domestic applications (Piehl et al., 2018; Rochman et al., 2019). The high prevalence of black MPs may reflect the degradation of black plastic waste or the extensive use of of black plastic products in both commercial and agricultural settings (Fan et al., 2023) (Fig. 5).

### Polymer composition

**Fig. 6:**
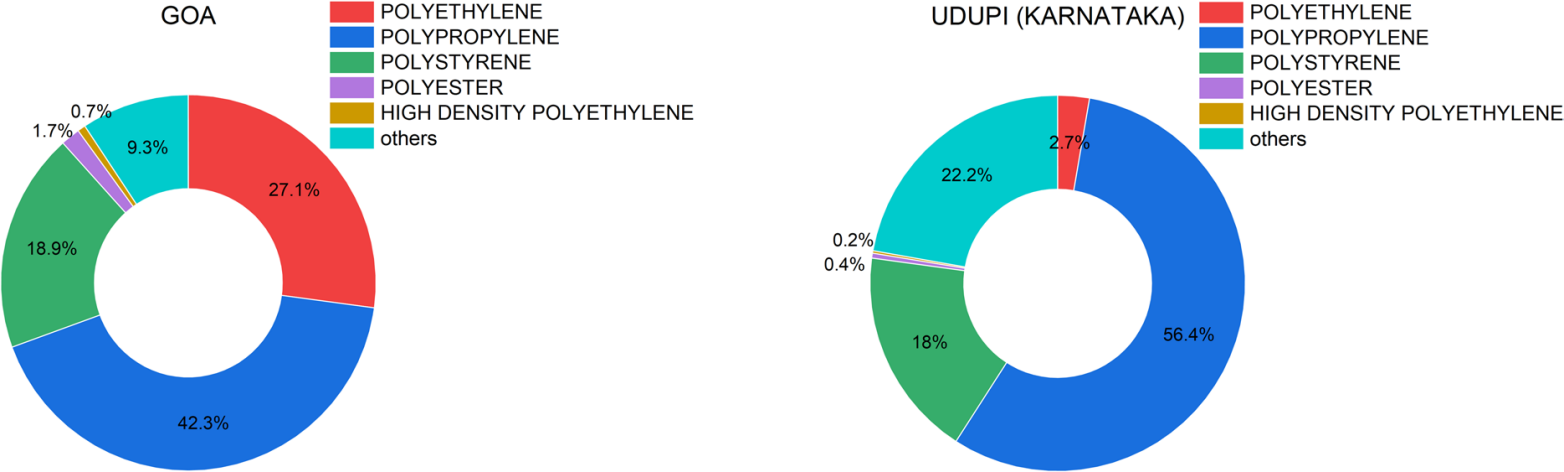
Polymer composition of MPs identified in agricultural soils from Goa and Udupi (Karnataka), presented as pie charts based on ATR-FTIR analysis. In Goa, the dominant polymers were polypropylene (42.3%), followed by polyethylene (27.1%), polystyrene (18.9%), and polyester (9.3%). In contrast, Udupi samples showed a higher dominance of polypropylene (56.4%), with lower proportions of polystyrene (22.2%), polyester (18%), and polyethylene (2.7%). Minor components included high-density polyethylene (HDPE) and other unidentified polymers

Microplastic particles in the 1–5 mm and 0.3–1 mm size range were analysed for polymer composition using Attenuated Total Reflectance Fourier Transform Infrared Spectroscopy (ATR-FTIR). Across both Goa and Udupi, the detected polymers included polypropylene (PP), polyethylene (PE), polystyrene (PS), polyesters, and high-density polyethylene (HDPE) (Fig. 6). Polypropylene was the most abundant polymer, accounting for 42.3% of MPs in Goa and 56.4% in Udupi. This was followed by PE, PS, and polyesters, while HDPE occurred in the lowest proportions (1% in Goa; 0.2% in Udupi). Additionally, unidentified polymers—likely arising from advanced degradation or analytical interference—represented 9% of the total in Goa and 22% in Udupi.

The dominance of PP and PE is consistent with their widespread agricultural use, particularly as plastic mulch films valued for durability, soil-moisture conservation, and weed suppression (Kasirajan & Ngouajio, 2012). Polystyrene, commonly used in food packaging, may enter agricultural soils through littering, atmospheric fallout, or incorporation via organic amendments (Feldman, 2008). The detection of polyester points to synthetic textile inputs, potentially originating from wastewater irrigation or airborne deposition. Identifying polymer types provides crucial insight into probable sources, environmental persistence, and potential ecological hazards of MPs in agricultural systems, forming the basis for targeted mitigation measures and more sustainable land-management practices.

### Surface morphology and elemental composition of microplastics

**Fig. 7:**
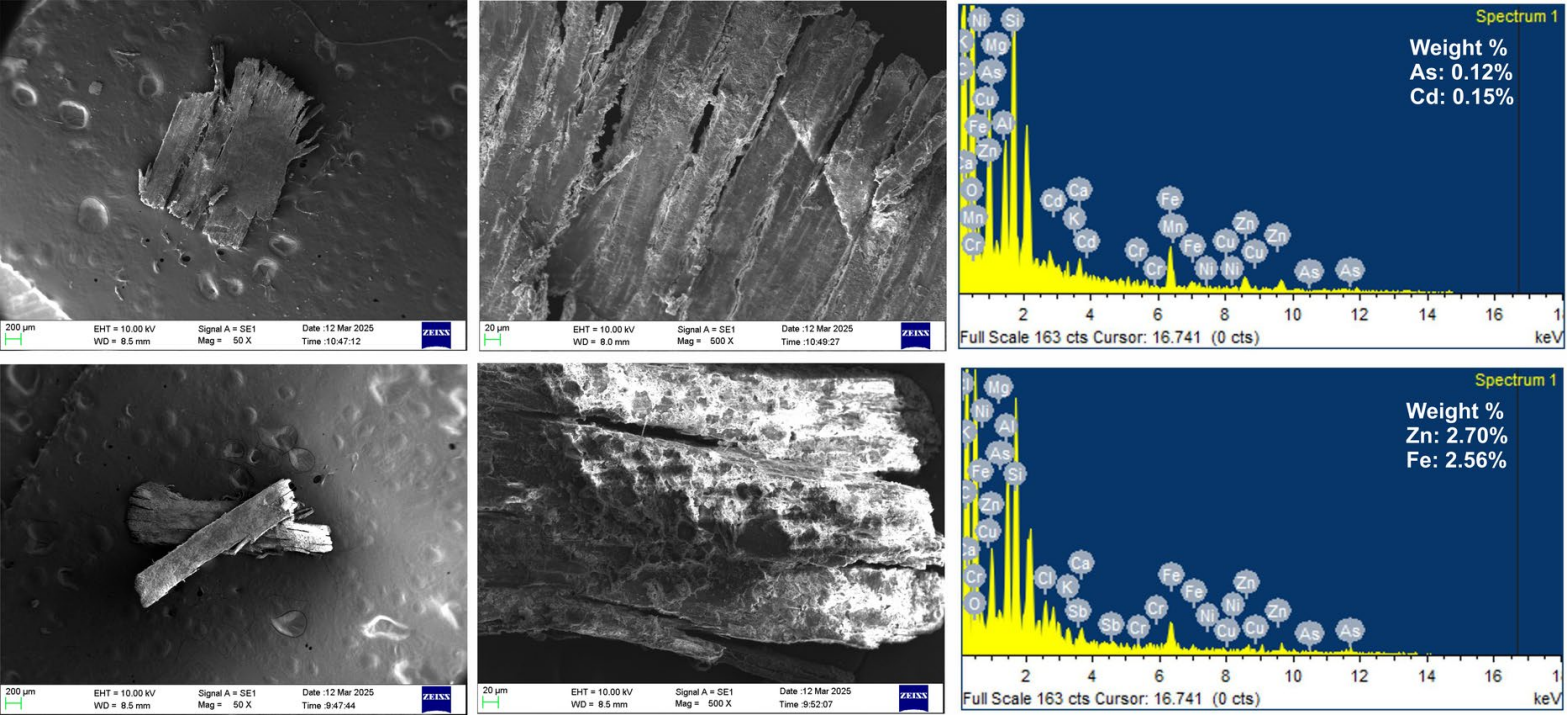
Scanning Electron Microscopy (SEM) images with Energy Dispersive X-ray Spectroscopy (EDS) showing the surface morphology and elemental composition of selected MP particles isolated from agricultural soils. The SEM micrographs reveal features such as cracks, pits, and surface abrasions, indicative of environmental weathering and degradation. EDS spectra confirm the elemental signatures commonly associated with plastic polymers and surface-adsorbed materials

Scanning Electron Microscopy (SEM) was used to characterize the surface features of selected MP particles, providing detailed insights into their morphology and degradation state. SEM images revealed films with hetereogenous surface textures (Fig. 7). Smooth, intact surfaces observed on some particles suggest recent environmental introduction with minimal weathering. In contrast, rough, fractured, and flaky surfaces indicate prolonged environmental exposure and degradation, consistent with physical abrasion and oxidative weathering (Borah et al., 2024).

Energy Dispersive X-ray Spectroscopy (EDS) analysis detected several heavy metals, including Nickel (Ni), Zinc (Zn), Arsenic (As), Cadmium (Cd), Chromium (Cr), Iron (Fe), and Antimony (Sb) (Fig. 7). Metals such as Fe and Cd are commonly used in inorganic pigments, suggesting intentional incorporation during plastic manufacturing(Khaleel et al., 2022). The presence of As likely reflects agrochemical contamination from historical or ongoing pesticide and herbicide use.

These results highlight the dual role of MPs as both physical pollutants and vectors for hazardous trace metals, potentially enhancing their mobility and bioavailability in soils (Holmes et al., 2012). Once incorporated into the soil matrix, such metals may be taken up by soil organisms, posing risks to soil health and enabling trophic transfer within terrestrial food webs (Wang et al., 2020).

### Risk assessment of microplastics

The Polymer Hazard Index (PHI) was used to evaluate the chemical toxicity risk associated with different polymer types in agricultural soils. In Goa, the highest PHI value reached 1859.9, corresponding to Hazard Level V—the highest category in the classification system proposed by Lithner et al. (2011). This elevated PHI reflects the substantial presence of high-risk polymers such as polyamide (PA) and polystyrene (PS), which fall under hazard levels IV and V and significantly influence the overall risk profile (Ranjani et al., 2021).

The Coefficient of Microplastic Impact (CMPI) was applied to assess ecological risk based on microplastic morphology. Distinct regional differences were observed. In Udupi, fibres dominated across all sites, with CMPI values exceeding 0.8, indicating an extreme potential impact on soil structure and function. Films exerted a moderate effect, while fragments showed minimal impact (CMPI < 0.1). In contrast, Goa displayed a more balanced distribution of morphotypes, with fibres, films, and fragments all registering moderate impact levels (CMPI 0.11–0.50).

The pronounced dominance of fibrous MPs in Udupi likely originates from synthetic textile fibres and wastewater sludge, both recognised sources of persistent fibres in soils. Such fibres can entangle with soil particles and biota, alter microbial activity, and modify porosity and aeration (Zubris & Richards, 2005), thereby potentially impairing soil health and fertility.

### Limitations of the study and implications for soil and crop health

This study offers important baseline insights into microplastic (MP) contamination in coastal agricultural soils; however, several limitations must be acknowledged. First, the relatively limited number of sampling sites restricts the generalizability of the findings across broader agricultural regions. Expanding spatial coverage and including a wider range of agroecological zones in future studies would enhance the representativeness and policy relevance of the data. Second, the absence of a pristine, undisturbed reference site (e.g., natural forest or grassland) limits the ability to establish baseline MP levels and to isolate the influence of agricultural practices on MP contamination. Inclusion of such control sites in future research would allow for a more robust evaluation of anthropogenic impacts. Third, key soil physicochemical parameters—such as pH, organic matter content, texture, and cation exchange capacity—were not assessed in the current study due to logistical constraints. These factors are known to influence the retention, transport, and degradation of MPs in soil systems. Their omission limits the understanding of MP behavior and interactions within the soil matrix. Microplastics in agricultural soils are increasingly recognized as a significant threat to soil quality and crop productivity. Originating from sources such as plastic mulching, sewage sludge, irrigation with contaminated water, and atmospheric deposition, these persistent particles can remain in soils for extended periods, disrupting key processes essential for healthy plant growth. Their diverse morphologies—fibres, films, and fragments—can interfere with natural soil aggregation, thereby altering porosity, bulk density, and water retention. Such changes may create less favourable conditions for root development and aeration (de Souza Machado et al., 2018). In certain cases, MPs can bind with soil particles and obstruct the movement of water and nutrients, reducing their bioavailability to plants. Furthermore, the potential uptake of MPs and their associated contaminants by crops raise concerns for food safety and human health (Li et al., 2020). These impacts highlight the urgent need for further research into the ecological and agronomic consequences of MP contamination in terrestrial systems.Recent studies indicate that MPs can accumulate on root surfaces, forming a physical barrier that impedes nutrient and water uptake. This interference may induce physiological stress in plants, manifested as reduced photosynthetic activity and stunted growth (Chang et al., 2024). Over time, such disruptions can lead to lower crop yields and diminished food quality. Although the severity of these impacts varies with MP type and concentration, current evidence suggests that their presence in soil is far from benign. Given that agricultural systems are already under pressure from climate change and land degradation, mitigating MP pollution is critical to safeguarding long-term soil health and ensuring sustainable crop production.

## Conclusion

This study investigated the prevalence, distribution, and characteristics of microplastics (MPs) in the agricultural soils of paddy fields in Goa and Udupi, two key rice-producing regions along India’s southwestern coast. The consistent detection of MPs in both surface and subsurface layers underscores the growing threat of plastic pollution in terrestrial ecosystems. Likely sources include urban runoff, atmospheric deposition, plastic mulching sheets, sewage sludge, and other anthropogenic inputs. A notable finding was the dominance of small-sized MPs, which can alter soil nutrient dynamics by influencing nutrient retention, microbial activity, and nutrient bioavailability to plants. The concurrent presence of heavy metals, as revealed by SEM–EDS analysis, points to a complex contamination scenario with potential adverse effects on soil health, crop productivity, and soil biota.

These results emphasize the need for standardized monitoring protocols and baseline assessments of MP contamination in agricultural soils. Incorporating regular surveillance within existing soil health monitoring frameworks would enable early detection and informed land management. Regulatory measures should focus on reducing plastic-based agricultural input such as mulch films, seed coatings, and polymer-based fertilizers—while promoting wastewater pre-treatment and filtration for irrigation. Farmer-focused educational initiatives are also essential to raise awareness of the long-term environmental risks of synthetic plastic use and to encourage the adoption of biodegradable alternatives and circular economic practices. Proactive implementation of these strategies is critical to safeguarding soil health, supporting sustainable food production, and mitigating the escalating risks of MP pollution in terrestrial ecosystems.

## Supplementary Information

Below is the link to the electronic supplementary material.

Supplementary file1 (DOCX 69.1 KB)

## Data Availability

All the data related to the manuscript is made available in Supplementary files.
