# Supplementary material for: Baseline assessment of microplastic contamination in agricultural soils from the coastal stretches of Karnataka and Goa, Southwestern India
Source: Environ Monit Assess. 2025 Aug 28;197(9):1053. doi: 10.1007/s10661-025-14513-5 (PMC12394264; doi:10.1007/s10661-025-14513-5)
Supplement: Supplementary file 1 — Supplementary file1 (DOCX 69.1 KB) [file 10661_2025_14513_MOESM1_ESM.docx]

**Baseline assessment of microplastic contamination in agricultural soils from the coastal stretches of Karnataka and Goa, Southwestern India**

Mahreen Lohani^1^, Ashwathi.C^1^ and Anish Kumar Warrier^1^

Department of Civil Engineering, Manipal Institute of Technology, Manipal, Karnataka, India.

Supplementary Table 1: Location of all the sampling sites

| SR.No | LOCATION ID | LATITUDE/ LONGITUDE |
| --- | --- | --- |
| 1 | (U)DUPI U1 | N13°22’27.396”  E74°46’52.338” |
| 2 | U2 | N13°22’27.066”  E74°46’52.146” |
| 3 | U3 | N13°22’32.556”  E74°46’2.61” |
| 4 | U4 | N13°22’34.188”  E74°45’59.268” |
| 5 | U5 | N13°22’4.806”  E74°47’40.338” |
| 6 | U6 | N13°22’9.258”  E74°47’49.734” |
| 7 | U7 | N13°22’10.656”  E74°47’47.802” |
| 8 | (G)OA G1 | N15°28’33.276”  E73°48’56.526” |
| 9 | G2 | N15°28’6.336”  E73°49’10.488” |
| 10 | G3 | N15°28’1.326”  E73°48’58.95” |
| 11 | G4 | N15°33’6.648”  E73°47’19.884” |
| 12 | G5 | N15°33’32.256”  E73°47’24.534” |
| 13 | G6 | N15°33’19.08”  E73°47’21.834” |
| 14 | G7 | N15°33’11.148”  E73°48’56.73” |
| 15 | G8 | N15°33’52.608”  E73°48’24.906” |

Supplementary Table 2: Total Microplastic Abundance in agricultural soils of Udupi.

| SAMPLE ID | FILM | FIBER | FRAGMENT | TOTAL |
| --- | --- | --- | --- | --- |
| U1 | 1 | 23 | 0 | 24 |
| U2 | 1 | 26 | 0 | 27 |
| U3 | 0 | 23 | 0 | 23 |
| U4 | 9 | 35 | 4 | 48 |
| U5 | 0 | 28 | 3 | 31 |
| U6 | 0 | 15 | 0 | 15 |
| U7 | 0 | 20 | 0 | 20 |
| U1,10CM | 0 | 36 | 0 | 36 |
| U2,10CM | 3 | 22 | 0 | 25 |
| U3,10CM | 2 | 32 | 0 | 34 |
| U4,10CM | 6 | 20 | 0 | 26 |
| U5,10CM | 2 | 15 | 1 | 18 |
| U6,10CM | 0 | 4 | 0 | 4 |
| U7,10CM | 1 | 8 | 2 | 11 |
| U1,20CM | 0 | 16 | 0 | 16 |
| U2,20CM | 2 | 13 | 0 | 15 |
| U3,20CM | 1 | 10 | 0 | 11 |
| U4,20CM | 4 | 14 | 1 | 19 |
| U5,20CM | 1 | 16 | 0 | 17 |
| U6,20CM | 0 | 10 | 1 | 11 |
| U7,20CM | 1 | 10 | 0 | 11 |
| U1,30CM | 0 | 5 | 0 | 5 |
| U2,30CM | 2 | 0 | 0 | 2 |
| U3,30CM | 3 | 3 | 0 | 6 |
| U4,30CM | 3 | 1 | 0 | 4 |
| U5,30CM | 0 | 4 | 1 | 5 |
| U6,30CM | 5 | 4 | 0 | 9 |
| U7,30CM | 2 | 1 | 1 | 4 |

Supplementary Table 3: Total Microplastic Abundance in agricultural soils of Goa.

| **Sample ID** | **FILM** | **FIBRE** | **FRAGMENT** | **WHOLE TOTAL** |
| --- | --- | --- | --- | --- |
| G1 | 8 | 55 | 6 | 69 |
| G2 | 35 | 33 | 7 | 75 |
| G3 | 12 | 15 | 5 | 32 |
| G4 | 3 | 20 | 2 | 25 |
| G5 | 6 | 16 | 0 | 22 |
| G6 | 4 | 17 | 1 | 22 |
| G7 | 5 | 13 | 5 | 23 |
| G8 | 3 | 22 | 2 | 27 |
| G1,10CM | 5 | 16 | 0 | 21 |
| G2,10CM | 26 | 24 | 7 | 57 |
| G3,10CM | 17 | 4 | 11 | 32 |
| G4,10CM | 0 | 13 | 1 | 14 |
| G5,10CM | 7 | 16 | 2 | 25 |
| G6,10CM | 6 | 44 | 1 | 51 |
| G7,10CM | 5 | 29 | 0 | 34 |
| G8,10CM | 3 | 22 | 0 | 25 |
| G1,20CM | 7 | 58 | 4 | 69 |
| G2,20CM | 100 | 30 | 50 | 180 |
| G3,20CM | 17 | 8 | 5 | 30 |
| G4,20CM | 5 | 3 | 2 | 10 |
| G5,20CM | 4 | 9 | 1 | 14 |
| G6,20CM | 5 | 9 | 1 | 15 |
| G7,20CM | 4 | 4 | 0 | 8 |
| G8,20CM | 1 | 5 | 0 | 6 |
| G1,30CM | 12 | 5 | 2 | 19 |
| G2,30CM | 13 | 4 | 7 | 24 |
| G3,30CM | 23 | 12 | 2 | 37 |
| G4,30CM | 8 | 2 | 3 | 13 |
| G5,30CM | 10 | 3 | 0 | 13 |
| G6,30CM | 3 | 3 | 0 | 6 |
| G7,30CM | 10 | 5 | 0 | 15 |
| G8,30CM | 8 | 0 | 0 | 8 |


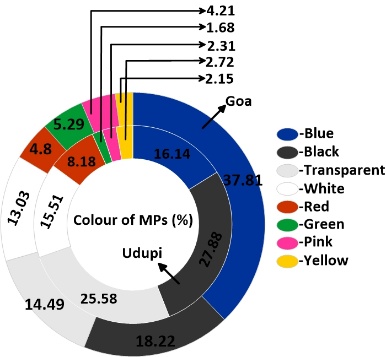


Supplementary figure 1: Total percentage of MPs by color present in Goa and Udupi.

Supplementary Table 4: Goa polymer count

| Polymer | Count |
| --- | --- |
| Polypropylene | 432 |
| Polyethylene | 277 |
| Polystyrene | 193 |
| Polyester | 17 |
| High density polyethylene | 7 |
| Others | 95 |

Total MPs 1021

Supplementary Table 5: Udupi polymer count

| Polymer | Count |
| --- | --- |
| Polypropylene | 269 |
| Polyethylene | 13 |
| Polystyrene | 86 |
| Polyester | 2 |
| High density polyethylene | 1 |
| Others | 106 |

Total MPs 477
